# Knowledge mapping of application of image-guided surgery in prostate cancer: a bibliometric analysis (2013–2023)

**DOI:** 10.1097/JS9.0000000000001232

**Published:** 2024-03-04

**Authors:** Na Zeng, Jian-Xuan Sun, Chen-Qian Liu, Jin-Zhou Xu, Ye An, Meng-Yao Xu, Si-Han Zhang, Xing-Yu Zhong, Si-Yang Ma, Hao-Dong He, Shao-Gang Wang, Qi-Dong Xia

**Affiliations:** Department and Institute of Urology, Tongji Hospital, Tongji Medical College, Huazhong University of Science and Technology Wuhan, People’s Republic of China

**Keywords:** Bibliometrics, CiteSpace, image-guided surgery, prostate cancer, VOSviewer

## Abstract

**Background:**

Image-guided surgery (IGS) refers to surgery navigated by medical imaging technology, helping doctors better clarify tumor boundaries, identify metastatic lymph nodes and preserve surrounding healthy tissue function. Recent studies have provided expectable momentum of the application of IGS in prostate cancer (PCa). The authors aim to comprehensively construct a bibliometric analysis of the application of IGS in PCa.

**Method:**

The authors searched publications related to application of IGS in PCa from 2013 to 2023 on the web of science core collection (WoSCC) databases. VOSviewer, CiteSpace, and R package ‘bibliometrix’ were used for bibliometric analysis.

**Results:**

Two thousand three eighty-nine articles from 75 countries and 2883 institutions led by the United States were included. The number of publications related to the application of IGS in PCa kept high in the last decade. Johns Hopkins University is the top research institutions. Journal of Nuclear Medicine has the highest popularity as the selection of journal and co-cited journal. Pomper Martin G. had published the most paper. Ali Afshar-Oromieh was co-cited most frequently. The clinical efficacy of PSMA-PET/CT in PCa diagnosis and treatment are main topics in this research field, with emerging focuses on the use of fluorescence imaging guidance technology in PCa. ‘PSMA’ and ‘PET/CT’ are the main keywords as long-term research hotspots.

**Conclusion:**

This study is the first bibliometric analysis of researches on application of IGS in PCa with three recognized bibliometric software, providing an objective description and comprehensive guidance for the future relevant investigations.

## Introduction

Highlights

- It is the first bibliometric analysis related to the application of image-guided surgery in prostate cancer.
- Three recognized bibliometric software, including VOSviewer, CiteSpace, and R package ‘bibliometrix’ were used.
- This study provides an objective description and comprehensive guidance for the future investigations on the application of image-guided surgery in prostate cancer.
- Data availability is not applicable to this review as no new data were created or analyzed in this study.

Prostate cancer (PCa) is the most frequently diagnosed cancer in over one-half (112 of 185) of the countries around the world and the fifth leading cause of cancer death among men, with an estimated 1.4 million new cases and 375 000 deaths worldwide in 2020^1^. As a complex and heterogenous cancer, several treatment options are available for PCa patients based on the cancer risk stage^2^. For early localized PCa, operations based on irradiation or current which only destruct tumors are available^3^, such as local radiotherapy, irreversible electroporation, and high-frequency irreversible electroporation (H-FIRE), etc., but radical prostatectomy (RP) is the only surgical treatment for localized PCa, as well as the optimal treatment of men with high-risk, clinically localized PCa^2,4^.

However, the radical excision of gland necessary and the possible occurrence of lymph node metastasis of PCa result in two postoperative management stresses^5^. One is the loss of patients’ postoperative functional outcomes attributing to the total prostate excision. And the other is the rate of positive surgical margins (PSMs) and biochemical recurrence cannot be negligible, which results from the inability to identify the boundaries of the tumor and potential metastases in lymph nodes and other organs. Despite rapid development in surgical approaches, traditional open radical prostatectomy was gradually substituted by laparoscopic radical prostatectomy (LRP) and robot-assisted laparoscopic prostatectomy (RALP) with similar oncological and better functional outcomes due to less invasion and more precision^6–8^. However, how to further clarify the boundaries of PCa and the presence of metastatic lymph nodes is still an open question owing to its feature of often multifocal occurrence with no-capsular infiltration. In addition to the experience and skills of operators, better surgical effect is in need of a keen eye better than naked eyes for distinguishing from the tumor and normal tissues. Therefore, the use of image-guided surgery (IGS) in PCa surgery has emerged^5^.

IGS is broadly defined as any surgeries assisted with the integration of stereotactic systems with advanced imaging and personal computers, compensating for the not-enough precision of naked eyes^9^. In PCa, the goals of IGS can be summarized as clarifying tumor boundaries, identifying metastatic lymph nodes and preserving surrounding healthy tissue as far as possible^10^, to decrease the rate of positive surgical margins (PSMs) and organ functional damage. Here, put simply, IGS was regarded as surgery assisted, guided and evaluated by preoperative and intraoperative medical images, benefiting tumor residues reduction, recurrence avoidance, and patients’ prognosis improvement.

Trans-rectal ultrasound-guided biopsy and MRI were traditional choices for PCa location but with the disadvantage of invasion and low precision, respectively^2^. With the development of imaging technology, positron emission tomography/computed tomography (PET/CT) particularly with radiolabeled choline is widely applied in preoperative PCa imaging. Prostate-specific membrane antigen (PSMA) is a specific target for PCa and highly expressed on the surface of PCa cells compared to normal prostate cells, coming to the most popular target of fluorescence probes^11^. Combined with ^68^Ga-PSMA, PET/CT performed well in the detection and intraprostatic localization of both primary and metastatic PCa^12,13^ before RP. Furthermore, the introduction and development of the Da Vinci surgical system in urology makes fluorescence imaging guided surgery more convenient^14,15^, providing a new strategy of immediate intraoperative guidance with real-time imaging feedback.

Several studies have demonstrated the feasibility of using indocyanine green (ICG), one mature fluorescent molecular, for real-time sentinel lymph node identification and lymph node dissection based on the near-infrared fluorescence (NIRF) imaging system^16–18^. Multiform fluorescence probes are also in active investigation as a result of concerns over inadequate half-life and poor tumor targeting ability of ICG^10^. Fu *et al*.^19^ designed a novel small-molecule fluorescence probe targeting prostate-specific membrane antigen (PSMA) for the surgical navigation of PCa. Additionally, nanobubbles and extracellular vesicles (EVs) are current star carriers to achieve targeting drug delivery^20^.

Bibliometrics is a literature analysis method that explores literature system and bibliometric characteristics of publications by mathematical, statistical, and other econometric methods^21^. It can provide bibliometric relationship of authors, organizations, countries, and references in relevant research field. Common bibliometric tools including VOSviewer^22^, CiteSpace^23^, and R package ‘bibliometrix’^24^ are widely applied in medical fields^25^. In 2019, Derks *et al*.^26^ comprehensively demonstrated recent preclinical and clinical advances in PSMA-targeted fluorescence-guided surgery of PCa. van Leeuwen *et al*.^27^ systematically discussed the application of IGS for managing lymphatic metastases in PCa. However, there is no quantitative and qualitive introduction of the application of IGS in PCa. What are the current high-profile imaging technologies? Is there any new fresh practice in this field? In this study, bibliometric analysis was used to reveal the objective performance and development of application of IGS in PCa from 1 January 2013 to 1 November 2023, aiming to provide a better insight of this research trends and look forward to future development prospects.

## Methods

### Search strategy

We conducted a literature search on the Web of Science Core Collection (WoSCC) database (https://www.webofscience.com/wos/woscc/basic-search), consisting of several databases with great influence and high authority, including the Science Citation Index (SCI) and Social Science Citation Index (SSCI), etc. The time spans are limited from 1 January 2013 to 1 November 2023. The search strategy consists of two parts: one is the retrieval of IGS (search term is #1, TS=(‘fluorescence’ OR ‘fluorescence-guided surgery’ OR ‘image guided surgery’ OR ‘molecular imaging’ OR ‘optical imaging’ OR ‘near-infrared fluorescence’ OR ‘fluorescence guided surgery’ OR ‘molecular imaging’ OR ‘hybrid imaging’ OR ‘fluorescent tracer’ OR ‘targeted fluorescent tracers’ OR ‘activatable tracers’)). The other is the limit of PCa (search term is #2, TS=(‘Prostate Neoplasms’ OR ‘Neoplasms, Prostate’ OR ‘Neoplasm, Prostate’ OR ‘Prostate Neoplasm’ OR ‘Neoplasms, Prostatic’ OR ‘Neoplasm, Prostatic’ OR ‘Prostatic Neoplasm’ OR ‘Prostate Cancer’ OR ‘Cancer, Prostate’ OR ‘Cancers, Prostate’ OR ‘Prostate Cancers’ OR ‘Cancer of the Prostate’ OR ‘Prostatic Cancer’ OR ‘Cancer, Prostatic’ OR ‘Cancers, Prostatic’ OR ‘Prostatic Cancers’ OR ‘Cancer of Prostate’ OR ‘Prostate Cancer’). The final search term is #1 AND #2. Only documents with ‘articles’ and ‘review’ type and written by English were included. Publications with other types and languages were excluded. Besides, we had glanced over all the included studies and artificially excluded those obviously unrelated to this field.

### Data analysis

VOSviewer (version 1.6.20) is a program available to construct and view bibliometric maps and extract key information from numerous publications^22^, often used to build authors or journals co-citation relationships and keyword co-occurrence relationships^28^. In this article, the software was used to analyze relationships among authors, countries, institutions, journals, references, and keywords.

CiteSpace (version 6.2.R4) is another application supporting visual exploration with knowledge mapping in bibliographic databases^23,29^. Here, CiteSpace was applied to display the trend of annual number of publications related to application of IGS in PCa, map the dual-map overlay of journals and to analyze reference with Citation Bursts.

The R package ‘bibliometrix’ (version 4.0.0) (https://www.bibliometrix.org) assisted to the thematic evolution analysis and construct a global distribution network of publications on the application of IGS in PCa^24^. The quartile and impact factor of the journal are obtained from Journal Citation Reports 2023.

All quantitative analysis of publication was completed by Microsoft Office Excel 2019. The exact functions and parameters used in these tools were provided in Supplementary File (Supplemental Digital Content 1, http://links.lww.com/JS9/C44).

## Results

### Quantitative analysis of publication

**Figure 1:**
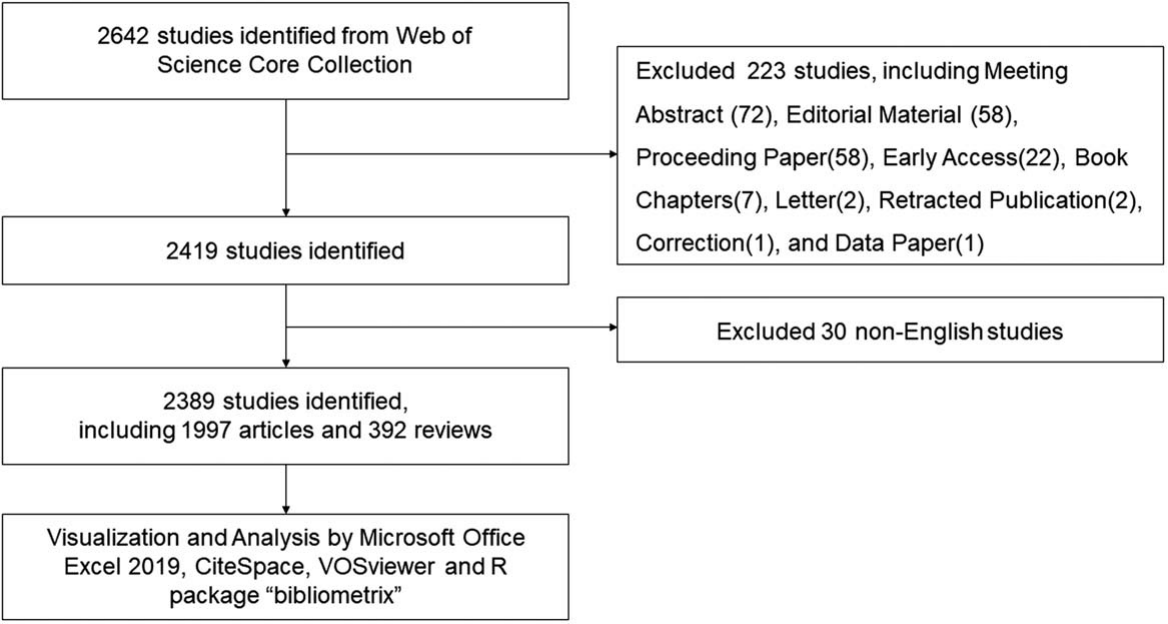
Publications screening flowchart.

**Figure 2:**
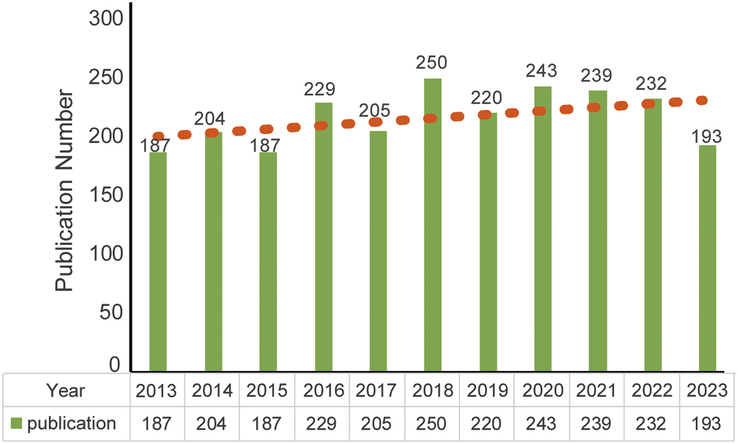
Annual output of research of the application of image-guided surgery in prostate cancer.

Based on search terms, a total of 2642 studies were identified form WoSCC, and meeting abstracts, editorial materials, and letters were excluded shown in Figure 1. Finally, a total of 2389 studies of application of IGS in PCa were collected including 1997 articles and 392 reviews. The exact quantity of publications of each year was provided in Figure 2. During the last decade, the number of publications related to the application of IGS in PCa has kept high, showing long-term attention on this topic. From 2013 to 2023, 250 researches were published in 2018, occupying the first place.

### Country and institutional analysis

**Figure 3:**
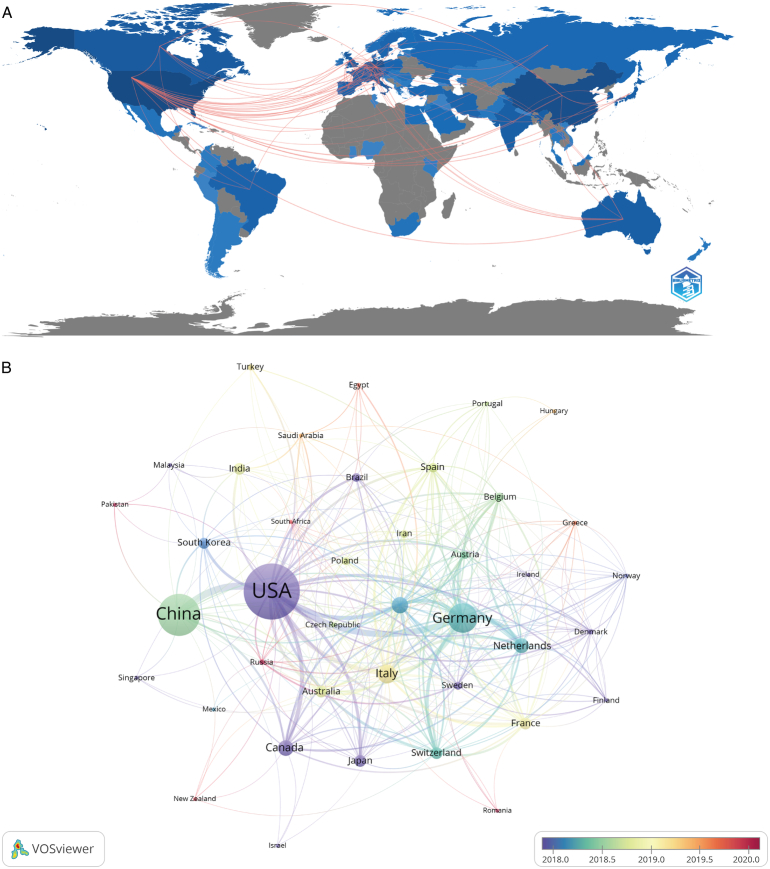
The geographical distribution (A) and visualization of countries (B) on research of application of image-guided surgery in.

These publications ranged from 75 countries and 2883 institutions, among which the top 10 countries are distributed mainly in Europe (*n*=6) (Table 1). The combined number of publications from the top three countries accounted for over half of the total (*n*=1730, 51.4%), composed of The United States (*n*=860, 25.5%) in North America, China (*n*=548, 16.3%) in Asia, and Germany (*n*=322, 9.6%) in Europe. Countries with the number of publications more than or equal to five were filtered and visualized used VOSviewer. The collaborative network in each country was provided in Figure 3, based on the number and relationships of publications. The United States has active collaboration with lots of countries including China, Germany, England, South Korea, Japan, Netherland, and Brazil. Germany displays close relationship with Netherlands, Australia, and Italy. Besides, the item’s color of the country represented the year with the highest number of publications from 2013 to 2023 (Fig. 3B). For example, the purple circle of the United States pointed out that the peak period of research publication was the end of 2017 and the early 2018.

**Table 1:** Top 10 countries and institutions on research of application of image-guided surgery in prostate cancer.

Rank	Country	Counts	Institution	Counts
1	The United States (North America)	860 (25.5%)	Johns Hopkins University (The United States)	77 (2.4%)
2	China (Asia)	548 (16.3%)	Technical University of Munich (Germany)	56 (1.8%)
3	Germany (Europe)	322 (9.6%)	Memorial Sloan Kettering Cancer Center (The United States)	52 (1.6%)
4	Italy (Europe)	167 (5.0%)	Leiden University (Netherlands)	43 (1.4%)
5	The Unite Kingdom (Europe)	135 (4.0%)	University of California, Los Angeles (The United States)	42 (1.3%)
6	Canada (North America)	120 (3.6%)	University of California, San Francisco (The United States)	42 (1.3%)
7	Netherlands (Europe)	115 (3.4%)	Case Western Reserve University (The United States)	39 (1.2%)
8	Australia (Oceania)	83 (2.5%)	Stanford University (The United States)	38 (1.2%)
9	France (Europe)	83 (2.5%)	University Medical Center Hamburg-Eppendorf (Germany)	35 (1.1%)
10	Switzerland (Europe)	80 (2.4%)	German Cancer Research Center (Germany)	32 (1.0%)

**Figure 4:**
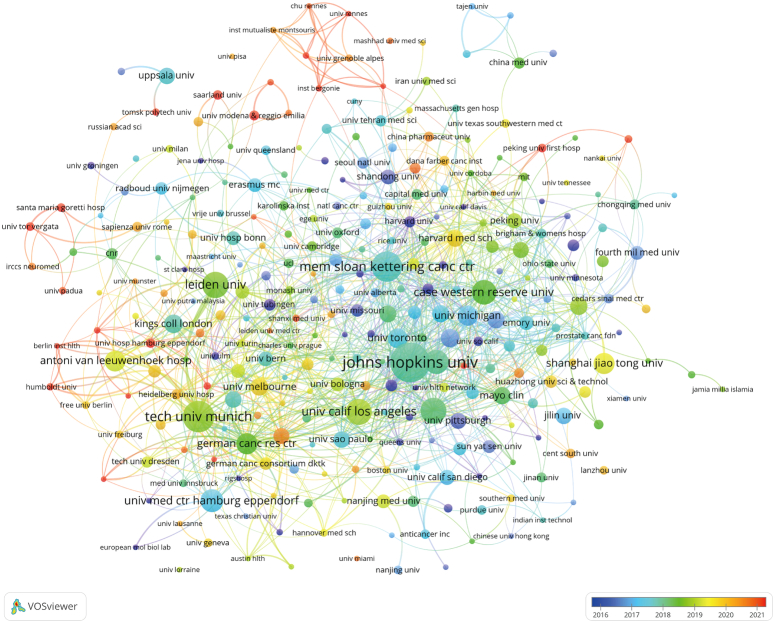
The visualization of institutions on application of image-guided surgery in prostate cancer.

The United States, Germany and Netherlands dominates the top 10 institutions on research publications with three-fifths of them located in the United States. The top three institutions are Johns Hopkins University (*n*=77, 2.4%), Technical University of Munich (*n*=56, 1.8%), and Memorial Sloan Kettering Cancer Center (*n*=52, 1.6%). Figure 4 provided the collaborative relationship among 292 institutions with more than five publications. Interestingly, Johns Hopkins University has close cooperation with following seven top institutions, but less connection with University Medical Center Hamburg-Eppendorf and German Cancer Research Center, which two are active cooperated with each other.

### Journals and co-cited journals

**Figure 5:**
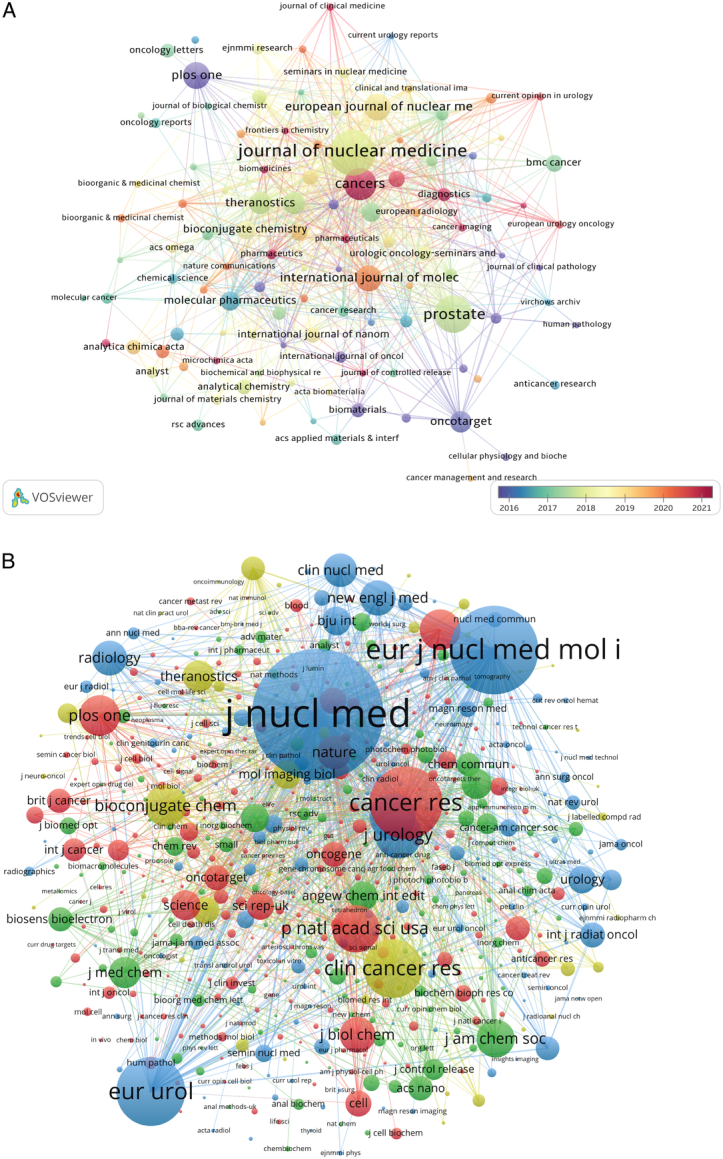
The visualization of journals (A) and co-cited journals (B) on research of application of image-guided surgery in prostate.

Seven hundred fifty-seven journals were searched about researches on application of IGS in PCa. Shown in Table 2, Journal of Nuclear Medicine published most papers (*n*=78, 5.8%), followed by Prostate (*n*=59, 4.4%), and Cancers (*n*=48, 3.5%). Theranostics (*n*=29, 2.1%, ranking seventh) has the highest impact factor (IF=12.4, Q1) among the top 15 journals, followed by Journal of Nuclear Medicine (IF=9.3, Q1). Notably, Oncotarget (*n*=27, 2.0%, ranking ninth) had been dropped by SCI in 2018. Removed journals with the number of relevant papers smaller than 5113 journals were included in bibliometric analysis (Fig. 5A). Journal of Nuclear Medicine has been cited most frequently from 2018 to 2019, and has large amounts of citations of Prostate and Cancers, etc.

**Table 2:** Top 15 journals and co-cited journals for research of application of image-guided surgery in prostate cancer.

Rank	Journal	Count	IF	Q	Co-cited Journal	Co-citation	IF	Q
1	Journal of Nuclear Medicine	78 (5.8%)	9.3	Q1	Journal of Nuclear Medicine	7024	9.3	Q1
2	Prostate	59 (4.4%)	2.8	Q2	European Journal of Nuclear Medicine and Molecular Imaging	4004	9.1	Q1
3	Cancers	48 (3.5%)	5.2	Q2	Cancer Research	3039	11.2	Q1
4	European Journal of Nuclear Medicine and Molecular Imaging	37 (2.7%)	9.1	Q1	European Urology	2806	23.4	Q1
5	PLoS One	37 (2.7%)	3.7	Q2	Clinical Cancer Research	2255	11.5	Q1
6	International Journal of Molecular Sciences	35 (2.6%)	5.6	Q1	Proceedings of the National Academy of Sciences of the United States of America	1647	11.1	Q1
7	Theranostics	29 (2.1%)	12.4	Q1	Journal of Urology	1587	6.6	Q1
8	Scientific Reports	28 (2.1%)	4.6	Q2	Journal of Clinical Oncology	1578	45.3	Q1
9	Oncotarget	27 (2.0%)	NA	NA	Bioconjugate Chemistry	1358	4.7	Q1
10	Molecular Imaging and Biology	23 (1.7%)	3.1	Q2	PLoS One	1335	3.7	Q2
11	Molecular Pharmaceutics	23 (1.7%)	4.9	Q1	Prostate	1331	2.8	Q2
12	Bioconjugate Chemistry	22 (1.6%)	4.7	Q1	Journal of the American Chemical Society	1195	15	Q1
13	BMC Cancer	19 (1.4%)	3.8	Q2	Journal of Biological Chemistry	1095	4.8	Q2
14	Frontiers in Oncology	18 (1.3%)	4.7	Q2	New England Journal of Medicine	1079	158.5	Q1
15	Molecules	18 (1.3%)	4.6	Q2	Radiology	1062	19.7	Q1

Table 2 also provided the top co-cited journals calculated by VOSviewer. All journals were cited more than 1000 times. Journal of Nuclear Medicine occupies the first place with 7024 co-citations, followed by European Journal of Nuclear Medicine and Molecular Imaging (co-citation=4004), Cancer Research (co-citation=3039), European Urology (co-citation=2806) and Clinical Cancer Research (co-citation=2255). Additionally, the impact factor of New England Journal of Medicine is the highest (IF=158.5), followed by Journal of Clinical Oncology (IF=45.3). Figure 5B displayed a co-citation network of 812 journals with at least 20 co-citations. Journal of Nuclear Medicine has tight co-citation relationships with European Journal of Nuclear Medicine and Molecular Imaging, and Cancer Research, etc.

**Figure 6:**
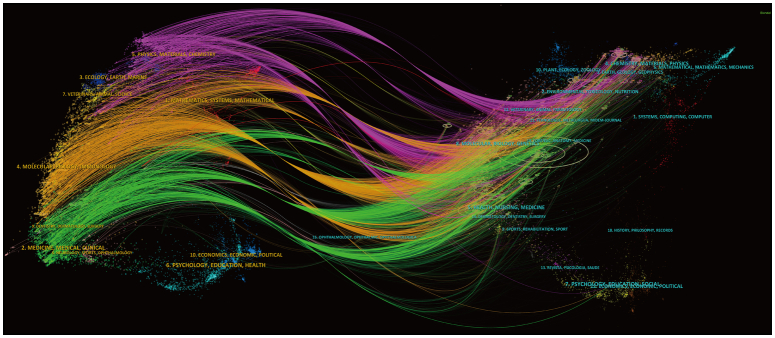
The dual-map overlay of journals on research of application of image-guided surgery in prostate cancer.

The citation relationships between journals and co-cited journals were drawn in the dual-map overlay (Fig. 6) of CiteSpace. The left clusters are citing journals and the right clusters are cited journals. Each label is centered at the cluster centroid of the corresponding journals and indicates corresponding disciplines in which citing articles were published. Each spline curve starts from a citing journal in the base map on the left and directs at a cited journal in the base map on the right^30^. As shown in Figure 6, the research published in Molecular/Biology/Genetics journals is mainly cited by literature in Molecular/Biology/Immunology journals. The research published in Health/Nursing/Medicine journals is mainly cited by literature in Medicine/Medical/Clinical journals.

### Authors and co-cited authors

**Figure 7:**
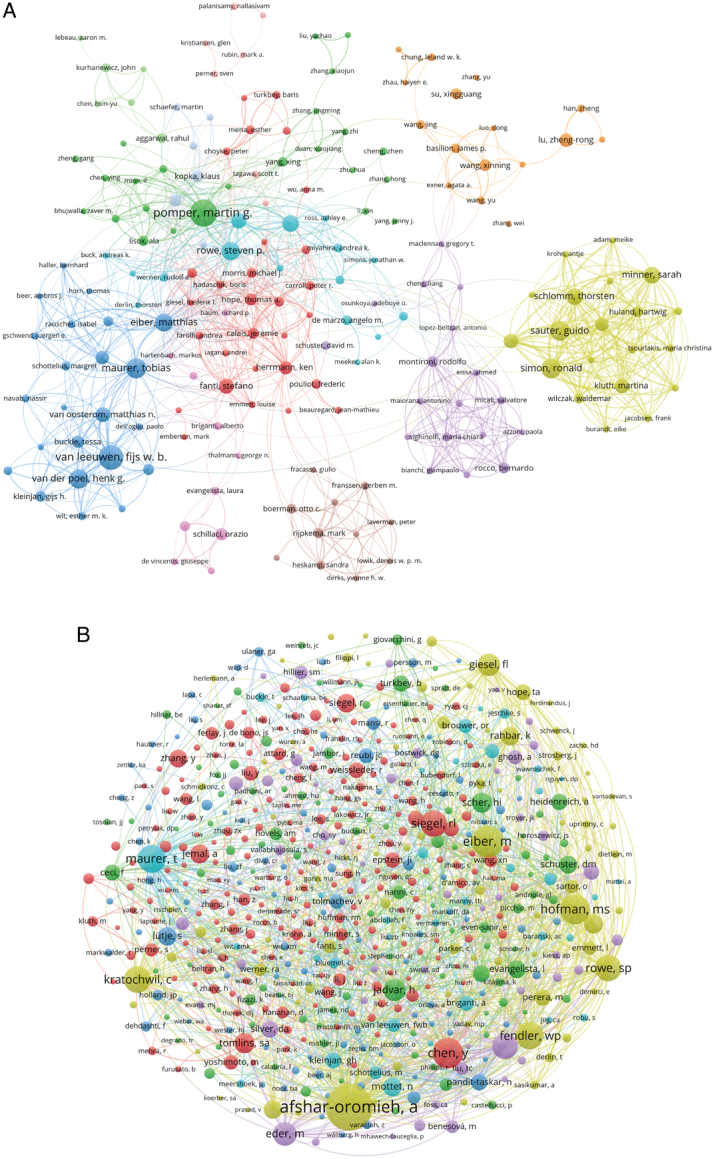
The visualization of authors (A) and co-cited Authors (B) on application of image-guided surgery in prostate cancer.

There were 14 044 authors involved the research on application of IGS in PCa. All of the top 10 authors had published at least 20 papers and Pomper Martin G. had published up to 40 papers, taking the first place in the left part of Table 3. We constructed a cooperative network based on 247 authors with at least five relevant publications (Fig. 7A) by VOSviewer. Articles with more than 25 authors had been automatically excluded. Close collaboration was observed among various authors. For example, Sauter Guido has positive association with Schlomm Thortsten, Simon Ronald, and Huland Hartwig, etc. shown with clusters in yellow.

**Table 3:** Top 10 authors and co-cited authors on research of application of image-guided surgery in prostate cancer.

Rank	Authors	Count	Co-cited authors	Citations
1	Pomper Martin G.	40	Afshar-Oromieh A	451
2	van Leeuwen Fijs W. B.	35	Eiber M^49^	259
3	Maurer Tobias	26	Chen Y	246
4	Sauter Guido	25	Hofman MS^33^	236
5	Simon Ronald	25	Maurer T^50^	215
6	van der Poel Henk G.	24	Fendler WP^77^	212
7	Rowe Steven P.	23	Kratochwil C	203
8	Eiber Matthias	22	Siegel RL^31^	200
9	Orlova Anna	22	Rowe SP^39^	197
10	Minner Sarah	20	Banerjee SR	189

A total of 59 758 co-cited authors were sorted and eight authors were co-cited no less than 200 times (Table 3). The top co-cited author is Afshar-Oromieh A (*n*=451), followed by Eiber M (*n*=259) and Chen Y (*n*=246). We mapped co-citation network graphs with 578 authors co-cited at least 20 times (Fig. 7B). Banerjee SR has active connection with multiple authors including Afshar-Oromieh A, Chen Y, Siegel RL, and Kratochwil C, Lvtje S, etc.

### Co-Cited references

**Figure 8:**
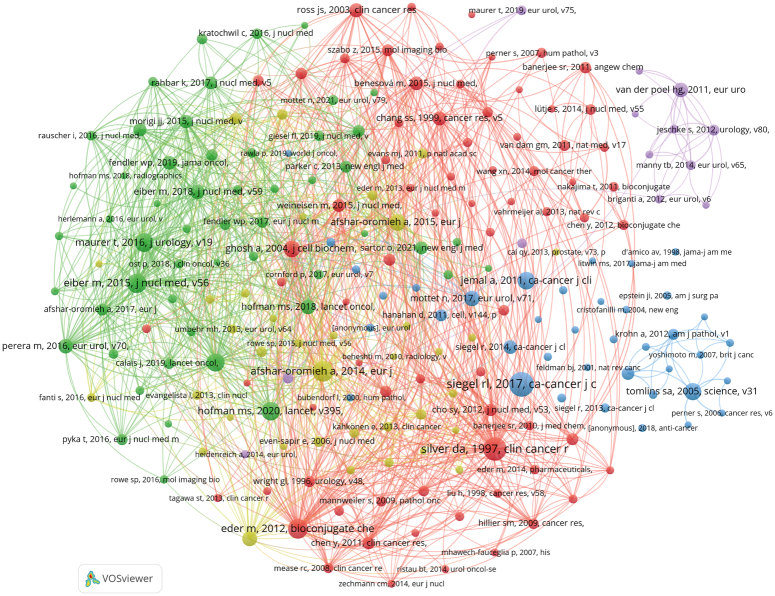
The visualization of co-cited references on research of application of image-guided surgery in prostate cancer.

There are 92 171 co-cited references on research of application of IGS in PCa in the last decade. Shown in Tables 4, two references were co-cited more than 100 times^31,32^. Two hundred fourteen references with at least 20 co-citations were filtered to the construction of the co-cited network map (Fig. 8). ‘Siver Da, 1997, Clin Cancer R’ displays active co-cited relationships with ‘Eder M, 2012, Boiconjugate Che’, ‘Afshar-Oromieh A and 2014, Eur J’, etc. Notably, ‘Siegal RL, 2017, Ca-Cancer J C’ shows no connection with any other items.

**Table 4:** Top 10 co-cited references on application of image-guided surgery in prostate cancer.

Rank	Co-cited reference	Main research content	Citations
1	Siegel RL, 2017, CA Cancer J Clin, V67, P7^31^	Cancer statistics based on data from 1930 to 2014 in the United States obtained from the National Center for Health Statistics (NCHS)	116
2	Silver DA, 1997, Clin Cancer Res, V3, P81^32^	Prostate-specific membrane antigen (PSMA) expression in normal and malignant human tissues	109
3	Afshar-Oromieh A, 2014, Eur J Nucl Med Mol I, V41, P11^34^	Comparison of PET imaging with a ^68^Ga-labeled PSMA ligand and ^18^F-choline-based PET/CT for the diagnosis of recurrent prostate cancer	92
4	Eder M, 2012, Bioconjugate Chem, V23, P688^48^	An introduction of ^68^Ga-complex lipophilicity and the targeting property of a urea-based PSMA inhibitor for PET imaging	85
5	Eiber M, 2015, J Nucl Med, V56, P668^49^	Evaluation of hybrid^68^Ga-PSMA ligand PET/CT in 248 patients with biochemical recurrence after radical prostatectomy	83
6	Maurer T, 2016, J Urology, V195, P1436^50^	Diagnostic efficacy of ^68^Gallium-PSMA positron emission tomography compared to conventional imaging for lymph node staging of 130 consecutive patients with intermediate to high-risk prostate cancer	80
7	Hofman MS, 2020, Lancet, V395, P1208^33^	A clinical trial to investigate whether novel imaging using PSMA PET/CT might improve accuracy and affect management	78
8	Hövels AM, 2008, Clin Radiol, V63, P387^51^	A meta-analysis about the diagnostic accuracy of CT and MRI in the staging of pelvic lymph nodes in patients with prostate cancer	76
9	Jemal A, 2011, CA Cancer J Clin, V61, P134^52^	Global cancer statistics based on GLOBOCAN 2008	72
10	Tomlins SA, 2005, Science, V310, P644^53^	An exploration of the significance of recurrent fusion of TMPRSS2 and ETS transcription factor genes in prostate cancer	70

### Reference with citation bursts

**Figure 9:**
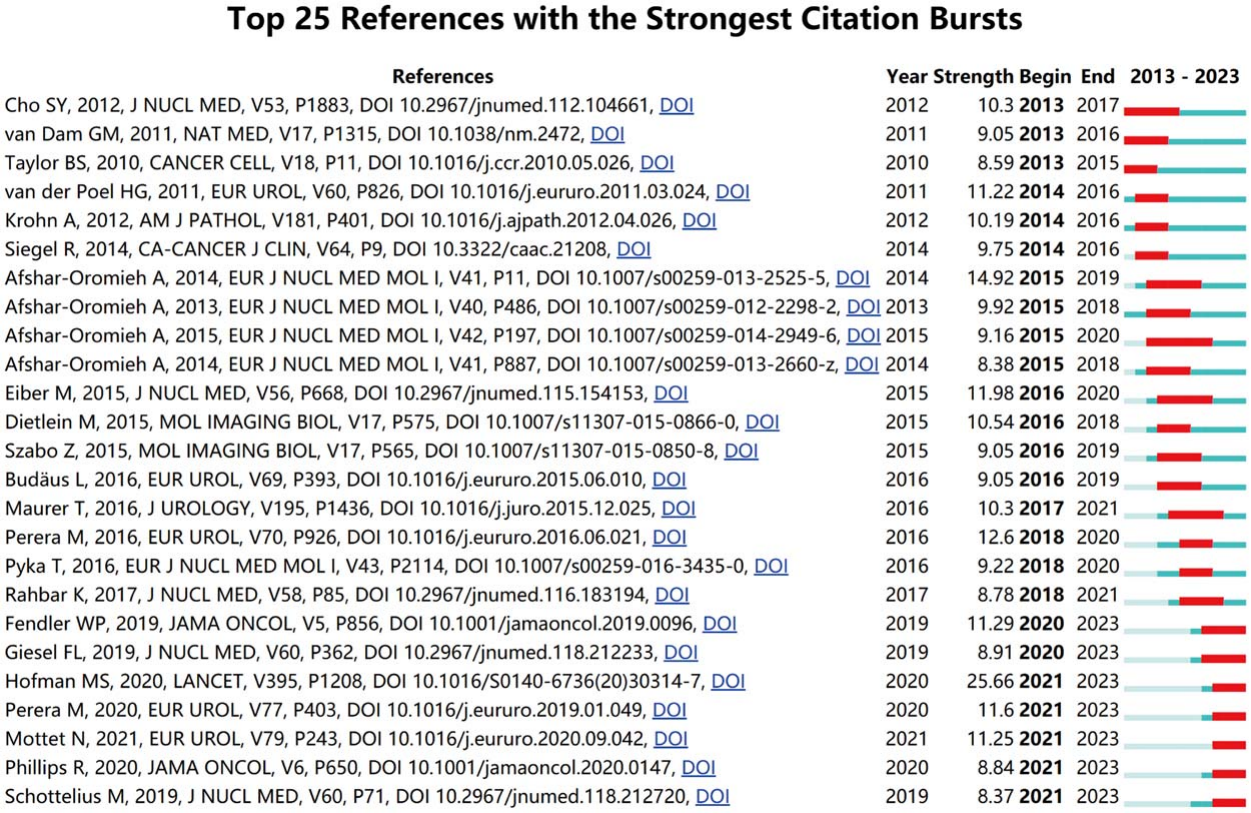
Top 25 references with strong citation bursts. A red bar indicates high citations in that year.

Reference with citation burst refers to papers with a surge in citation frequency after publication, indicating a high attention to relevant topics. Figure 9 provided top 25 references with the strongest citation bursts identified by CiteSpace. The red bar represents the period of strong citation burstiness during 2013 to 2023. Citation bursts for references appeared as early as 2013 and as late as 2021. The reference with the strongest citation burst (strength=25.66) ranked 21 in Table 5 and was titled ‘Prostate-specific membrane antigen PET-CT in patients with high-risk PCa before curative-intent surgery or radiotherapy (proPSMA): a prospective, randomised, multicenter study’, authored by Hofman *et al*.^33^ with citation bursts from 2021 to 2023. The reference with the second strongest citation burst (strength=14.92) was titled ‘Comparison of PET imaging with a ^68^Ga-labeled PSMA ligand and ^18^F-choline-based PET/CT for the diagnosis of recurrent PCa’^34^, which was also the third top co-cited reference on research of application of IGS in PCa. It was published in European Journal of Nuclear Medicine and Molecular Imaging by Ali Afshar-Oromieh *et al*. with 5-year citation bursts (2014–2019). Table 5 listed the detailed information about the 25 references in the order of the publications in Figure 9, showing a burst strength ranging from 8.37 to 25.66, and endurances strength ranging from 2 to 5 years.

**Table 5:** The main research contents of the 25 references with strong citations bursts.

Rank	Strength	Main research content
1	10.3	Biodistribution, tumor detection, and radiation dosimetry of ^18^F-DCFBC, a low-molecular-weight inhibitor of prostate-specific membrane antigen, in patients with metastatic prostate cancer^63^
2	9.05	The in-human results of intraoperative tumor-specific fluorescence imaging in ovarian cancer by folate receptor-α targeting^64^
3	8.59	Integrative genomic profiling of human prostate cancer^65^
4	11.22	Clinical proof of concept of an integrated functional imaging approach using a multimodal tracer for the intraoperative laparoscopic fluorescence guidance to the sentinel lymph node in prostate cancer patients^14^
5	10.19	Demonstration of the positive impact of genomic deletion of PTEN on tumor progression and early PSA recurrence in ERG fusion-positive and fusion-negative prostate cancer^66^
6	9.75	Cancer statistics based on data from 1930 to 2010 in the United States obtained from the National Center for Health Statistics (NCHS)^67^
7	14.92	Comparison of PET imaging with a ^68^Ga-labeled PSMA ligand and ^18^F-choline-based PET/CT for the diagnosis of recurrent prostate cancer^34^
8	9.92	The biodistribution of the ^68^Ga-gallium-labeled PSMA ligand used in PET imaging in humans and evaluation of tumor lesions for the diagnosis of prostate cancer^68^
9	9.16	The diagnostic value of PET/CT imaging with the ^68^Ga-labeled PSMA ligand HBED-CC in the diagnosis of recurrent prostate cancer^69^
10	8.38	Comparison of PET/CT and PET/MRI hybrid systems using a ^68^Ga-labeled PSMA ligand for the diagnosis of recurrent prostate cancer^70^
11	11.98	Evaluation of hybrid^68^ Ga-PSMA ligand PET/CT in 248 patients with biochemical recurrence after radical prostatectomy^49^
12	10.54	Comparison of ^18^F-DCFPyL and ^68^Ga-PSMA-HBED-CC for PSMA-PET imaging in patients with relapsed prostate cancer^71^
13	9.05	Initial evaluation of ^18^F-DCFPyL for PSMA-targeted PET imaging of prostate cancer^72^
14	9.05	Initial experience of ^68^Ga-PSMA PET/CT imaging in high-risk prostate cancer patients prior to radical prostatectomy^73^
15	10.3	Comparison of the diagnostic efficacy between ^68^Gallium-PSMA positron emission tomography and conventional imaging for lymph node staging of 130 consecutive patients with intermediate to high-risk prostate cancer^6^
16	12.6	A systematic review and meta-analysis of the sensitivity, specificity, and predictors of positive 68Ga-PSMA positron emission tomography in advanced prostate cancer^74^
17	9.22	Comparison of bone scintigraphy and ^68^Ga-PSMA PET for skeletal staging in prostate cancer^75^
18	8.78	A German multicenter study investigation of ^177^Lu-PSMA-617 radioligand therapy in advanced prostate cancer patients^76^
19	11.29	A prospective single-arm clinical trial to assess the ^68^Ga-PSMA-11 PET accuracy in localizing recurrent prostate cancer^77^
20	8.91	Detection efficacy of 18F-PSMA-1007 PET/CT in 251 patients with biochemical recurrence of prostate cancer after radical prostatectomy^78^
21	25.66	A clinical study of PSMA PET-CT in patients with high-risk prostate cancer before curative-intent surgery or radiotherapy (proPSMA)^33^
22	11.6	A systematic review and meta-analysis of ^68^Gallium PSMA positron emission tomography in advanced prostate cancer-updated diagnostic utility, sensitivity, specificity, and distribution of PSMA-avid lesions^79^
23	11.25	EAU-EANM-ESTRO-ESUR-SIOG Guidelines on Prostate Cancer-2020 Update. Part 1: Screening, Diagnosis, and Local Treatment with Curative Intent^80^
24	8.84	A RCT about the outcomes of observation vs stereotactic ablative radiation for oligometastatic prostate cancer^81^
25	8.37	Synthesis and preclinical characterization of the PSMA-targeted hybrid tracer PSMA-I&F for nuclear and fluorescence imaging of prostate cancer^82^

### Hotspots and frontiers

The co-occurrence analysis of keywords helps to capture the hotspots in the application of IGS in PCa, and the top 30 high-frequency keywords calculated by VOSviewer were summarized in Table 6. PET/CT and PSMA appeared more than 200 times, representing the major imaging technique and favorable tumor-target applied in the application of IGS in PCa. The appearances of nanoparticles (count=36), NIRF imaging (count=26), ICG (count=22), and peptides (count=22) in the list promote an emerging status of NIRF imaging technology in combination with ICG or other fluorescence molecules in the application of IGS in PCa.

**Table 6:** Top 30 keywords on research of application of image-guided surgery in prostate cancer.

Rank	Keywords	Counts	Rank	Keywords	Counts
1	prostate cancer	758	16	PSA	37
2	PET/CT	270	17	breast cancer	36
3	molecular imaging	268	18	nanoparticles	36
4	PSMA	210	19	prostate	36
5	fluorescence imaging	111	20	photodynamic therapy	35
6	apoptosis	98	21	autophagy	33
7	MRI	83	22	sentinel lymph node biopsy	31
8	cancer	74	23	radiotherapy	29
9	CT	52	24	cytotoxicity	28
10	theranostics	51	25	radical prostatectomy	27
11	imaging	42	26	near-infrared fluorescence imaging	26
12	image-guided surgery	39	27	biochemical recurrence	23
13	hybrid imaging	38	28	radiopharmaceuticals	23
14	optical imaging	38	29	ICG	22
15	biomarkers	37	30	peptides	22

**Figure 10:**
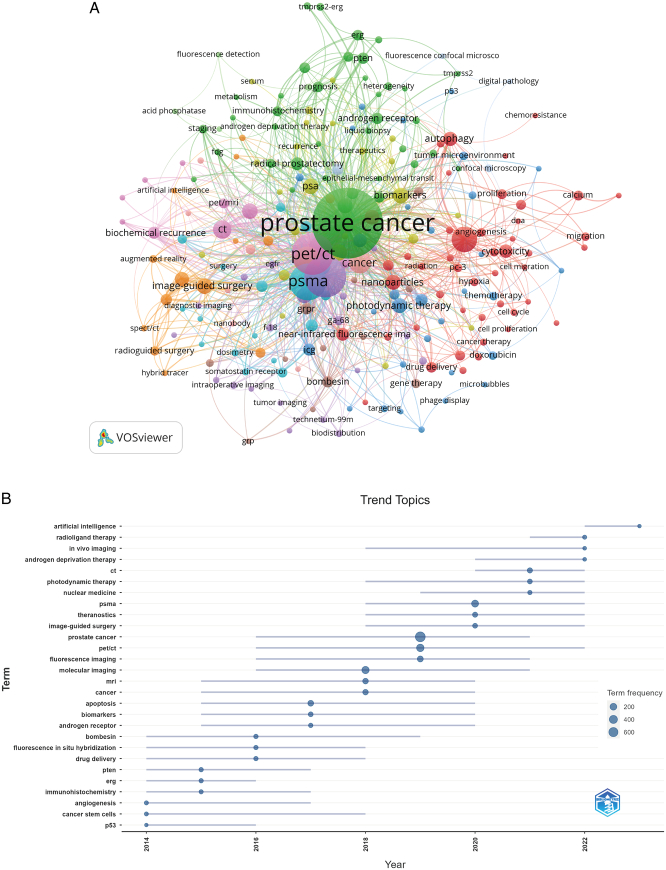
Keyword cluster analysis (A) and trend topic analysis (B).

We filtered author keywords occurring more than four times and created a co-occurrence bibliometric map with cluster analysis (Fig. 10A). Different colors represent different research directions and there were five major clusters performed in Figure 10A, respectively, colored mainly in green, red, orange, and pink.

The trend topic analysis of the keywords (Fig. 10B) displayed that from 2013 to 2018, the research in this period mainly focused on cancer stem cells, immunohistochemistry, apoptosis, and molecular imaging, etc., which reflected on a mechanism exploration of image-guidance. From 2019 to 2023, researchers have focused on PSMA as target and the clinical application of IGS in PCa, and the main keywords are PET/CT, photodynamic therapy, and in vivo imaging, etc. In addition, artificial intelligence has appeared frequently in 2023, indicating a huge assistance of artificial intelligence to the development of the application of IGS in PCa.

## Discussion

### General information

During the last decade, the amounts of researches published kept high, showing a long-term popularity of the application of IGS in PCa. The United States is the top country conducting relevant studies, followed by China and Germany. Sixty percent of the top 10 institutions are located in the United States followed by Germany (*n*=3, 30%) and Netherlands (*n*=1, 10%). The phenomenon may benefit from the leading scientific and economic strength of above countries, on which the development of IGS depends. Besides, with the spread of early PCa screening in above leading countries, there are more patients with early PCa badly in need of more precise and less injury treatment, which promoting the investigations of IGS in the United States, China and Germany.

Close relationships were observed among the United States, England, Netherlands, and Germany. Institutions of the United States and Germany have active collaborations with each other, including Johns Hopkins University, University of California, Los Angeles, Technical University of Munich, and German Cancer Research Centre. However, as the second top country, China has weak connection with other countries beside the United States, so do the institutions in China. Extensive cooperation among various countries and institutions is positively encouraged, which benefits a joint conquer of technological barriers and a comprehensive evaluation of technologies or drugs used in IGS based on multiple populations. Nevertheless, it is notable to standardize operators’ practice before formally carrying out the cooperation, since expertise is acknowledged as an interfering factor of surgery effect.

5.8% articles were published in Journal of Nuclear Medicine (IF=9.3, Q1) and 2.7% articles were published in European Journal of Nuclear Medicine and Molecular Imaging (IF=9.1, Q1), respectively, ranked first and forth in the list of top journals of application of IGS in PCa. Meanwhile, these two journals were the top two journal in the list of co-cited journals, tallying with the trend of much attention to the targeting molecules in IGS. Besides, relevant researches tend to be published mainly in three categories of journals based on the dual-map overlay: Medicine/Medical/Clinical, Molecular biology/immunology, and Physics/Materials/Chemistry. It is speculated that these three clusters in turn match relevant clinical trials for identifying the efficacy of IGS, investigations on the mechanism of imaging molecules and generation of corresponding surgical equipment.

In terms of authors, Pomper Martin G. and van Leeuwen Fijs W. B. published the most articles, the number of which was both greater than 30. Most researches of Pomper Martin G. were around the value of molecular imaging technique, especially PSMA-PET/CT for PCa^35–38^. Published in Ca-A Cancer Journal for Clinicians (IF=254.7, Q1) in 2022, one review written by Pomper Martin G. comprehensively supported the current application and clinical value of molecular imaging technique, pointing a promising future direction of IGS^39^. The latest researches of van Leeuwen Fijs W. B. had focused on the application of robotic surgery and fluorescence guidance in the treatment of PCa^40–44^. Two clinical researches^42,43^ expressed the optimistic prospective of fluorescence imaging-guided robot-assisted laparoscopic prostatectomy.

With regard to the co-cited authors, Afshar-Oromieh A was the most frequent co-cited author (citation=451), followed by Eiber M (citation=259). In 2015, Afshar-Oromieh A reported the first evaluation of the distribution of ^68^Ga-PSMA-617 in normal tissues and in PCa lesions as well as the radiation exposure by the radioligand in PET imaging^45^, which proposed that ^68^Ga-PSMA-617 was able to show lesions of PCa with high contrast. The next year, Afshar-Oromieh A published a review, systematically summarizing the value of PSMA ligands in diagnose and therapy of PCa^46^. Similarly, one clinical trials joint by Eiber M proved the value of ^68^Ga-PSMA-11 PET imaging for the detection of nodal metastases in men with intermediate- to high-risk PCa^47^. Obviously, these achievements have provided examples and laid experimental foundation for research of the application of IGS in PCa.

### Knowledge base

A co-cited reference is defined as one research that is cited together by multiple publications. Top 10 co-cited references were selected to clarify the research basis of application of IGS in PCa. Most studies paid attention to the properties and application value of PSMA and medical imaging technology, especially PSMA PET/CT in the diagnose and surgery of PCa^32–34,48–51^, representing the popularity of PSMA PET/CT in relevant field. The first and ninth top co-cited references were respectively cancer statistics of the United States^31^ and whole world^52^, confirming the domination of the United States in relevant research field and the follow-up of other countries. Notably, one research exploring key transcription factor genes in PCa^53^ ranked 10th in the list of top 10 co-cited references, probably because recurrent fusion of TMPRSS2 and ETS transcription factor genes is one of the common causes of PCa.

### Hotspots and frontiers

References with citation bursts reflect on the emerging topics in a particular field. According to the burst period (Fig. 9) and main contents of publications (Table 5), relevant researches mainly focused on specific molecules of PCa before 2016. Since 2016, the clinical efficacy PSMA-PET/CT in PCa diagnosis and treatment has always been the hot spots in the research of application of IGS in PCa, consistent with the current focus on the performance of PSMA in urology surgery^26^. Additionally, top 30 keywords (Table 6) agreed with above conclusion. Excluding words like PCa, molecular imaging, fluorescence imaging, and cancer, etc., the keywords mainly include PET/CT, PSMA, biomarkers, nanoparticles, near-infrared inflorescence imaging, and ICG, etc. Coupled with the trend of topic terms shown in Figure 10B, we concluded two suggestions on research of application of IGS in PCa.

On the one hand, the design and development of imaging agents play a dominant role in promoting the application of IGS in PCa. It is no doubt that the precise location of tumor is the foundation of applying image-guide technique in tumor resection. Previously, ICG is the most widely used imaging agents in PCa, which is feasible to determine the location of sentinel lymph nodes and guide the range of pelvic lymph node dissection, especially after the development of RALP or the Da Vinci robotic platform^14,54^. However, the poor target ability of ICG cannot be ignored^10^ and how to accurately target tumor cells remain to be the focus and obstacle of IGS. As a specific target for PCa cells, there have been several strategies for targeting PSMA in recent years, including PSMA antibodies, single chain antibodies (scFv), targeting peptides, and nucleic acid aptamers, etc. For example, ^177^Lu-PSMA-617 has been potential in the radiotherapy treatment of PCa^55^. ^68^Gallium PSMA-11 (^68^Ga PSMA-11) has been approved as the first drug for PET imaging of PSMA positive lesions in men with PCa by the U.S. Food and Drug Administration (FDA) in 1 December 2020^56^. That may explain scholars’ continued and wide attentions to PSMA. Moreover, OTL78, a PSMA-targeted NIR agent has been designed and under clinical trials recently^54,57^. On this basis, ICG with multiform PSMA-targeted transport system is under investigation, to make up its deficiency of short half-life and poor biocompatibility. For example, Sun JX *et al*.^20^ proposed a promising drug transport system composed of PSMA-targeted EVs and fluorescence molecules. Yixuan Wang *et al*.^58^ had synthesized PSMA-targeted nanobubbles loading ICG and made good imaging performance in mice. Furthermore, it is expectable that new targets, imaging molecules and carries with better biological features will be explored. Take STEAP1 as an example, it is more widely and specifically expressed than PSMA in metastatic castration-resistant prostate cancer (mCRPC)^59^. Various NIR fluorophore with better imaging ability than ICG is expected to identified. All of the above enrich the composition of drug transport system.

On the other hand, the development of complete set and technology of IGS should not be ignored. From traditional MRI to PET/CT with high sensitivity, technological advances have diversified the selection of locating PCa, which provide available development environment for possible future IGS^60^. Moreover, Da Vinci robotic platform equipped with NIRF imaging system have practically helped to streamline clinical utilization of real-time imaging during a surgery, making IGS in PCa technically feasible^18^, which is superior to PET/CT with advantages of no invasion, no radiation, clearer images, and real-time feedback, etc. It is wishful to search or design novel imaging agents based on present available imaging techniques. In addition, the potential of integration of artificial intelligence and computer aided visualization used in PCa surgery has been proved by several studies, including the cystoscopic identification of bladder tumors^61^ and predictive technologies for prostate biopsies^62^. Compared with the naked eye, the integration of artificial intelligence shows better performance in identifying minimal lesions and work efficiency. Someday this computing technology would be adapted and harnessed for real-time lymph node identification or even partial prostate tumor resection not just prostatectomy during fluorescence-guided prostatectomy. We are looking forward to a better combination between integration of artificial intelligence and image-guide surgery base on hardware improvement, algorithm modification, and interdisciplinary communication and cooperation enhancement.

To sum up, better targeting and imaging molecules and more advanced surgical equipment benefit the development of IGS in PCa, which depend on the progress on multidisciplinary fields, including not only medicine, but biochemistry, computer, and mechanism. Notably, related surgical paradigm and skill training for doctors should catch up with the development.

### Advantages and shortcomings

This study is the first bibliometric analysis of researches on application of IGS in PCa with three recognized bibliometric software, providing an objective description and comprehensive guidance for the future relevant investigations. However, there are some limitations. Firstly, the data of this study are only from the WoSCC database, which is not able to fully cover relevant publications. Secondly, non-English writing papers were excluded in this analysis, which may prejudice the evaluation of related papers. Thirdly, articles included are too many to fully screened so that there may be some unfitted articles confounding the analysis. Fourthly, only articles and reviews written by English were retrieved in our analysis, which also might result in incomplete data collection from other types of publications. Some valuable studies written by their mother tongue in non-English countries are also ignored. In addition, since the citations contained self-citations, for practical reasons, we did not account for self-citations in our analysis, which may contain some controversial literature. Lastly, some publications included by related database may be delayed so the citations and H-index have existing flaws.

## Conclusion

IGS has promising application aspects in PCa especially with the emerging development of NIRF imaging technology. The steady number of related studies over the last decade reveals the maintained value of the application of IGS in PCa worldwide. Up to date, the United States occupied the leading place in the promotion of application of IGS in PCa, followed close by China and Germany. The collaboration and connection among various countries and organizations remain to be strengthened. According to this analysis, scholars are devoted to design and develop more effective and safe imaging molecules with the introduction of NIRF imaging system in PCa surgery. Under the pursuit of precise and minimally invasive treatment of PCa, there are great advantages for the exploration of IGS in PCa. Notably, not only the development of new imaging molecules but the updating of supporting equipment should be paid attention.

## Ethical approval

This review does not deal with any original human or animal data.

## Consent

This review does not involve any patients or volunteers.

## Sources of funding

This work has no funding.

## Author contribution

N.Z. and J.-X.S.: analyzed the data, wrote the manuscript, and drew the figures; Q.-D.X. and S.-G.W.: designed the study; C.-Q.L., J.-Z.X., Y.A., M.-Y.X., and S.-H.Z.: contributed to the critical revision of the manuscript; X.-Y.Z., S.-Y.M., and H.-D.H.: contributed to the critical revision of figures and tables. All authors read and approved the final manuscript.

## Conflicts of interest disclosure

All authors declare no financial and personal relationships.

## Research registration unique identifying number (UIN)

It is not applicable for this review.

## Guarantor

Qi-Dong Xia and Shao-Gang Wang are the guarantors of this review.

## Data availability statement

All data generated or analyzed during this study are included in this published article and referenced articles are listed in the references section.

## Provenance and peer review

Not commissioned, externally peer-reviewed.

## Supplementary Material
