# Supplementary material for: Knowledge mapping of application of image-guided surgery in prostate cancer: a bibliometric analysis (2013–2023)
Source: Int J Surg. 2024 Mar 4;110(5):2992–3007. doi: 10.1097/JS9.0000000000001232 (PMC11093506; doi:10.1097/JS9.0000000000001232)
Supplement: Supplementary file 1 [file js9-110-2992-s001.docx]

**Supplementary File**

The exact functions and parameters used in VOSviewer, CiteSpace and R package “bibliometrix” are provided as follows in order of figures:

**Figure 3:**

**A.** R package “bibliometrix”: Social Structure -> Countries’ Collaboration World Map.

**B.** VOSviewer: countries with the number of publications less than 5 were excluded.

**Figure 4:**

VOSviewer: institutions with the number of publications less than 5 were excluded.

**Figure 5:**

**A.** VOSviewer: journals with the number of publications less than 5 were excluded.

**B.** VOSviewer: journals with the number of co-citations less than 20 were excluded.

**Figure 6:**

CiteSpace: Overlay Maps -> Add overlay -> import the data that we had run before

**Figure 7:**

**A.** VOSviewer: authors with the number of papers less than 5 were excluded. Articles with more than 25 authors had been automatically excluded.

**B.** VOSviewer: authors with the number of co-citations less than 20 were excluded.

**Figure 8:**

VOSviewer: references with the number of co-citations less than 20 were excluded.

**Figure 9:**

CiteSpace:

1. Settings: Term Type -> Burst Terms; Node Types -> Keyword.

2. GO -> Visualize.

3. Control Panel -> Burstness -> View.

**Figure 10:**

**A.** VOSviewer: keywords with the number of occurrences less than 5 were excluded.

**B.** R package “bibliometrix”: Words -> Trend Topics.
